# Supplementary material for: Tumorigenicity decrease in Bcl-xL deficient MDCK cells ensuring the safety for influenza vaccine production
Source: PLoS One. 2024 Dec 16;19(12):e0311069. doi: 10.1371/journal.pone.0311069 (PMC11649150; doi:10.1371/journal.pone.0311069)
Supplement: S1 File — (ZIP) [file pone.0311069.s002.zip › S2_File/4_KOG_enrichment/enrichmap/B_vs_A.sign_KOG_enrichment_map.pdf]

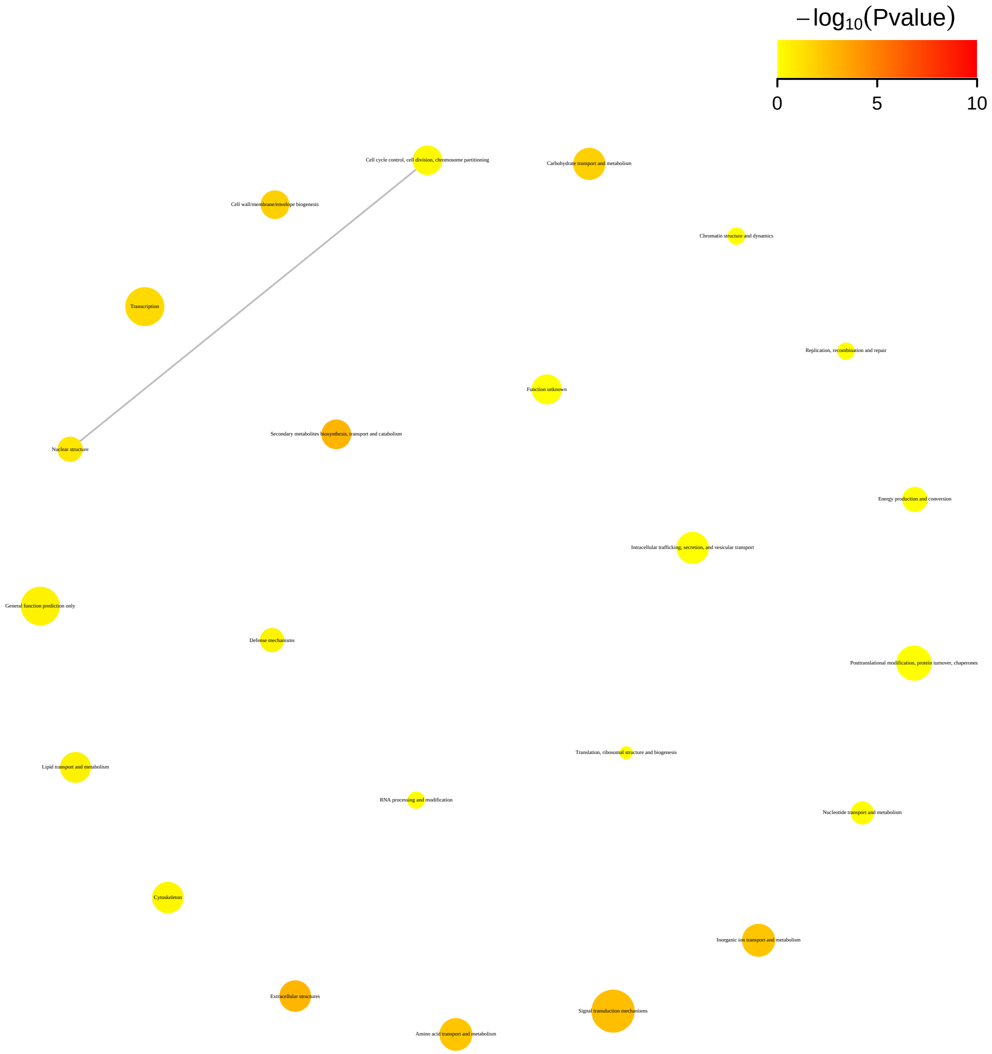

$-\log_{10}(\text{Pvalue})$
0
5
10
Cell cycle control, cell division, chromosome partitioning
Carbohydrate transport and metabolism
Cell wall/membrane/envelope biogenesis
Chromatin structure and dynamics
Transcription
Replication, recombination and repair
Function unknown
Secondary metabolites biosynthesis, transport and catabolism
Nuclear structure
Energy production and conversion
Intracellular trafficking, secretion, and vesicular transport
General function prediction only
Defense mechanisms
Posttranslational modification, protein turnover, chaperones
Translation, ribosomal structure and biogenesis
Lipid transport and metabolism
RNA processing and modification
Nucleotide transport and metabolism
Cytoskeleton
Inorganic ion transport and metabolism
Extracellular structures
Signal transduction mechanisms
Amino acid transport and metabolism
